# Characterization of complete mitochondrial genome in *Myuroclada maximowiczii* (G.G. Borshch.) Steere & W.B. Schofield

**DOI:** 10.1080/23802359.2021.1934161

**Published:** 2021-06-14

**Authors:** Yeong-Deok Han, Jin-Woo Jung, Youngeun Choi, Kwang-Bae Yoon, Young-Jun Yoon

**Affiliations:** a Research Center for Endangered Species, National Institute of Ecology, Yeongyang, Republic of Korea; b Department of Ecology Landscape Design, Jeonbuk National University, Jeonju, Republic of Korea

**Keywords:** *Myuroclada maximowiczii*, moss, mitochondrial genome

## Abstract

The mitogenome of *Myuroclada maximowiczii* (GenBank accession number MT834960) has a total length of 104,216 bp and encodes 40 protein-coding genes, three ribosomal RNAs, and 24 transfer RNAs. The overall nucleotide composition is asymmetric (29.6% A, 29.4% T, 21.2% G, and 19.8% C), with AT content (59.0%) higher than GC content (41.0%). The gene arrangement of this mitogenome is identical to that in other bryophytes. Phylogenetic trees were constructed using complete mitochondrial genome sequences of 24 bryophytes publicly available in GenBank and the mitogenome sequence derived in this study. Our phylogenetic analysis revealed that *M. maximowiczii* clustered in a clade with other hypnalean taxas.

The genus *Myuroclada* (Bryophyta, Brachytheciaceae) is represented in Korea by one taxon (Choe 1980). *Myuroclada maximowiczii* grows on the base of trees, on rocks, in soil, and on humus, and it is widely distributed in Korea, China, Japan, and Russia as well as Alaska (Noguchi 1988). Morphologically, *M. maximowiczii* resembles *Myurella julacea*; however, it differs by the smooth laminal cells and clearly costate leaves (Hu et al. 2008). In this study, the mitogenome of *M. maximowiczii* was assembled using Illumina sequencing data, which would be helpful for the phylogenetic studies of diverse lineage of mosses.

A fresh patch of *M. maximowiczii* was obtained from Yeongyang County, in Gyeongsangbuk Province (36° 38′ 50.41″ N; 129° 9′ 13.94″ E), on 13 January 2020. A specimen was deposited at Jeonbuk National University Herbarium (www.jbnu.ac.kr, Young-Jun Yoon, yjyoon@nie.re.kr) in Korea under the voucher number YYJ 20200105-1. Total cellular DNA was isolated from fresh thallus material using DNeasy^®^ Plant Mini Kit (Qiagen, Hilden, Germany). DNA library was constructed using TruSeq Nano DNA Library Prep Kit (Illumina, San Diego, CA) and then sequenced using HiSeq 2 × 150 bp Sequencing System (Illumina, San Diego, CA). Sequencing reads were trimmed and filtered using Trimmomatics v0.36 tools (Bolger et al. 2014) and FastQC v0.11.5 (Andrews 2010), respectively. Filtered reads were mapped to the *de novo* assembly using Geneious 8.1.9 (Biomatters, Auckland, New Zealand) and NOVOplasty 2.4 softwares (Dierckxsens et al. 2016).

The mitochondrial genome of *M. maximowiczii* (MT834960) is a circular molecule 104,216 bp long, with 59.0% AT consensus and the following base composition: A = 29.6%, T = 29.4%, G = 21.2%, and C = 19.8%. This mitochondrial genome encodes 40 protein-coding genes (PCGs), three ribosomal RNAs, and 24 transfer RNAs, and its gene arrangement is identical to that in other Hypnales. Of the 40 PCGs, 37 PCGs of *M*. *maximowiczii* mitogenome start with ATG codon, while three PCGs with CTG (*nad9* and *tatC*) and TTG (*atp1*) codons. All PCGs are stopped with the typical termination codon TAA, TAG, and TGA.

To confirm the phylogenetic affinities of *M. maximowiczii* within bryophytes, its mitogenome was sequenced and compared with complete mitochondrial genomes of 21 Bryophyta and three Marchantiophyta species obtained from GenBank. Concatenated sequence dataset was constructed by selecting 33 PCGs present in the mitogenome of all bryophytes for phylogenetic analysis (Goryunov et al. 2018), using Geneious 8.1.9. A phylogenetic tree was performed using the maximum likelihood method based on the Jones Taylor Thornton matrix model with 1000 bootstrap replicates in MEGA X software. *M. maximowiczii* is resolved, albeit with no support, as sister to *Anomodon rugelii* and *A. attenuatus*, and together with *Climacium* they compose a robust Hypnales (Figure 1). The mitogenome of *M. maximowiczii* is the first one assemble for the Brachytheciaceae and may provide a reference for phylogenetic studies of diverse lineage of mosses.

**Figure 1.**
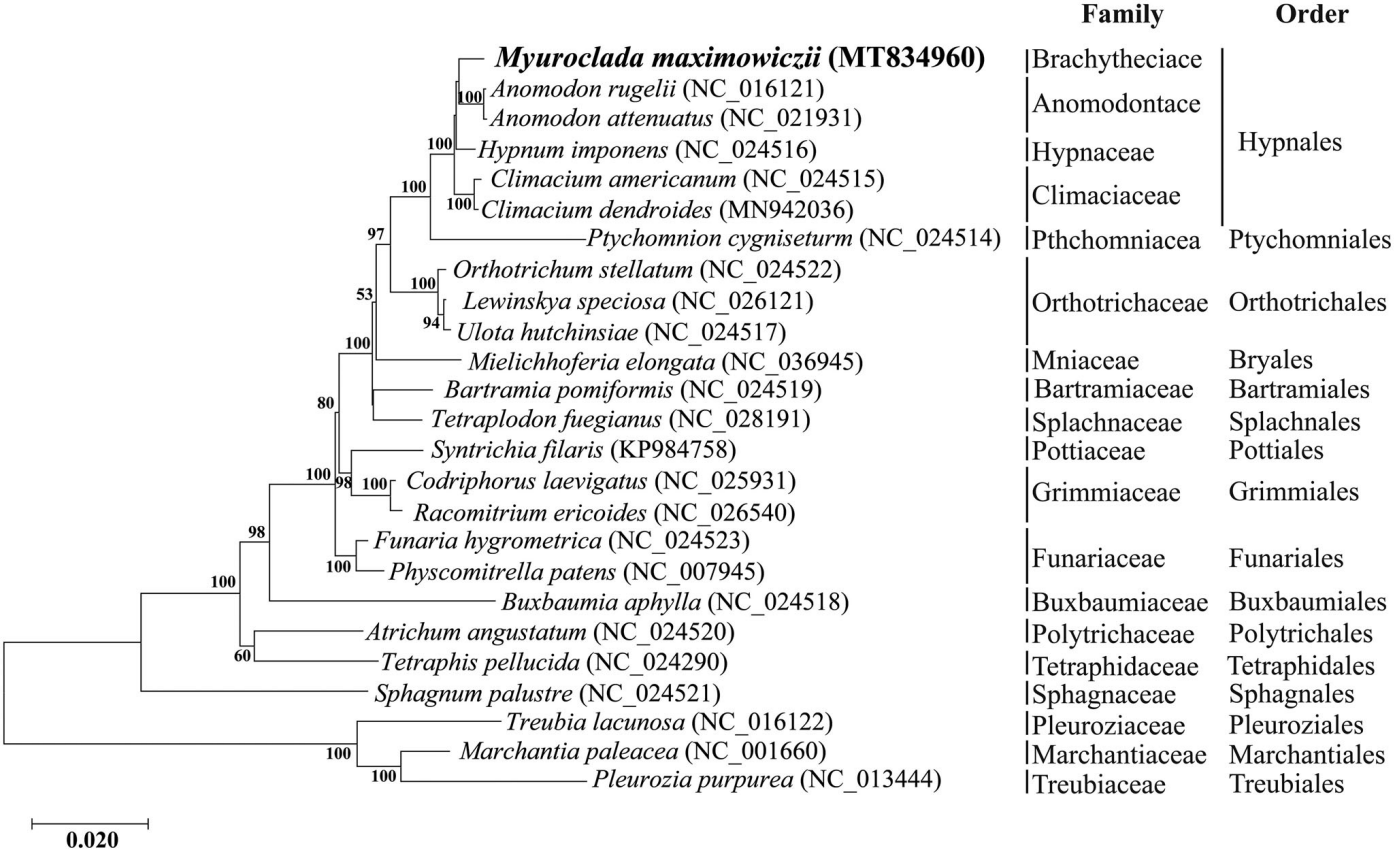
Phylogenetic relationship among *Myuroclada maximowiczii* constructed by maximum-likelihood analysis based on 33 mitochondrial protein-coding genes common in all taxa. A bootstrap values above 50% are given at the nodes. Three Marchantiophyta species were selected as outgroup.

## Data Availability

The complete mitogenome sequence can be accessed via accession no. MT834960 in GenBank of NCBI at https://www.ncbi.nlm.nih.gov. The associated BioProject, SRA, and Bio-Sample numbers are PRJNA698627, SAMN17730361, and SRR13605954, respectively.
